# Urinary Peptidomic Profiling In Post‐Acute Sequelae of SARS‐CoV‐2 Infection: A Case‐Control Study

**DOI:** 10.1002/pmic.70074

**Published:** 2025-11-21

**Authors:** Dilara Gülmez, Justyna Siwy, Katharina Kurz, Ralph Wendt, Miroslaw Banasik, Björn Peters, Emmanuel Dudoignon, Francois Depret, Mercedes Salgueira, Elena Nowacki, Amelie Kurnikowski, Sebastian Mussnig, Simon Krenn, Samuel Gonos, Judith Löffler‐Ragg, Günter Weiss, Harald Mischak, Manfred Hecking, Eva Schernhammer, Joachim Beige, UriCoV Working Group

**Affiliations:** ^1^ Department of Epidemiology Center for Public Health Medical University of Vienna Vienna Austria; ^2^ Mosaiques Diagnostics GmbH Hannover Germany; ^3^ Department of Internal Medicine II (Infectious Diseases, Immunology, Rheumatology, Pneumology) Medical University of Innsbruck Innsbruck Austria; ^4^ Division of Nephrology St. Georg Hospital Leipzig Germany; ^5^ Department of Nephrology Transplantation Medicine and Internal Diseases Wrocław Medical University Wroclaw Poland; ^6^ Department of Molecular and Clinical Medicine Institute of Medicine the Sahlgrenska Academy at University of Gothenburg Gothenburg Sweden; ^7^ Region Västra Götaland Skaraborg Hospital Department of Nephrology Skövde Sweden; ^8^ Department of Anaesthesiology and Critical Care Hôpital Saint Louis ‐ Lariboisière AP‐HP Universite Paris Cité Paris France; ^9^ Virgen Macarena Hospital University of Seville Seville Spain; ^10^ Université Des Patients – Sorbonne Université Paris France; ^11^ IUVABIT e.U. Analytics & Data Science Vienna Austria; ^12^ Department of Nephrology Medical University of Vienna Vienna Austria; ^13^ Kuratorium For Dialysis and Transplantation (KfH) Leipzig Leipzig Germany; ^14^ Department of Internal Medicine II Martin‐Luther‐University Halle‐Wittenberg Halle Germany

**Keywords:** biomarker, mass spectrometry, ME/CFS, PASC, urinary peptidomics

## Abstract

Post‐acute sequelae of severe acute respiratory syndrome coronavirus 2‐infection (PASC) is challenging to diagnose and treat, and its molecular pathophysiology remains unclear. Urinary peptidomics can provide valuable information on urine peptides that may enable improved and specified PASC diagnosis. Using standardized capillary electrophoresis‐MS, we examined the urinary peptidomes of 50 patients with PASC 10 months after COVID‐19 and 50 controls, including healthy individuals (*n* = 42) and patients with non‐COVID‐19‐associated myalgic encephalomyelitis/chronic fatigue syndrome (ME/CFS) (*n* = 8). Based on peptide abundance differences between cases and controls, we developed a diagnostic model using a support vector machine. The abundance of 195 urine peptides among PASC patients significantly differed from that in controls, with a predominant abundance of collagen alpha chains. This molecular signature (PASC195) effectively distinguished PASC cases from controls in the training set (AUC of 0.949 [95% CI 0.900–0.998; *p* < 0.0001]) and independent validation set (AUC of 0.962 [95% CI 0.897–1.00]; *p* < 0.0001]). In silico assessment suggested exercise, GLP‐1RAs and mineralocorticoid receptor antagonists (MRAs) as potentially efficacious interventions. We present a novel and non‐invasive diagnostic model for PASC. Reflecting its molecular pathophysiology, PASC195 has the potential to advance diagnostics and inform therapeutic interventions.

**Statement of Significance of the Study:**

Despite the recent emergence of omics‐derived candidates for post‐acute sequelae of SARS‐CoV‐2 infection (PASC), the pending validation of proposed markers and lack of consensus result in the continuous reliance on symptom‐based criteria, being subject to diagnostic uncertainties and potential recall bias. Building upon prior findings of renal involvement in acute COVID‐19 pathophysiology and PASC‐associated alterations, we hypothesized that the use of urinary peptides for PASC‐specific biomarker discovery, unlike conventional specimens that have been utilized thus far, may offer complementary information on putative disease mechanisms. In the present study, 195 significantly expressed peptides were used to form a classifier termed PASC195, which effectively discriminated PASC from non‐PASC (*p* < 0.0001), including healthy individuals and non‐COVID‐19‐associated myalgic encephalomyelitis/chronic fatigue syndrome, in both the derivation (*n* = 60) and an independent validation set (*n* = 40). The peptidome profile associated with PASC was consistent with a shift in collagen turnover, with most PASC195 peptides derived from alpha chains. Ongoing inflammatory responses, hemostatic imbalances, and endothelial damage were indicated by cross‐sectional variations in endogenous peptide excretion.

**Abbreviations**
A1AT: Alpha‐1‐antitrypsin
APOA2: Apolipoprotein A‐II
APOA4: Apolipoprotein A‐IV
ARB: Angiotensin II receptor blockers
AT III: Antithrombin‐III
AUC: Area under the curve
B2M: Beta‐2‐microglobulin
CALR: Calreticulin
CCC: Canadian Consensus Criteria
CE‐MS: Capillary‐electrophoresis‐mass‐spectrometry
CI: Confidence intervals
CLCB: Clathrin
CO1A1: Collagen alpha‐1(I)
CO1A2: Collagen alpha‐2(I)
CO3A1: Collagen alpha‐1(III)
CO4A2: Collagen alpha‐2(IV)
COEA1: Collagen alpha‐1(XIV)
COVID‐19: Coronavirus disease 2019
EBV: Epstein‐Barr virus
eGFR: Estimated glomerular filtration rate
EMRs: Electronic medical records
FIBA: Fibrinogen alpha
FIBB: Fibrinogen beta
GLP‐1RAs: Glucagon‐like peptide‐1 receptor agonists
GOLGA6L2: Golgin subfamily A member 6‐like protein 2
GSK3A: Glycogen synthase kinase‐3 alpha
IBS: Irritable bowel syndrome
IQR: Interquartile range
MASP2: Mannan‐binding lectin serine protease 2
ME/CFS: Myalgic encephalomyelitis/chronic fatigue syndrome
MRAs: Mineralocorticoid receptor antagonists
NHM: Delayed postural hypertension or neurally mediated hypotension
PASC: Post‐acute sequelae of SARS‐CoV‐2 infection
PEM: Post‐exertional malaise
PGBM: Basement membrane‐specific heparan sulfate proteoglycan core protein
POTS: Postural orthostatic tachycardia syndrome
ROC: Receiver operating characteristic
S100A8/A9: Calprotectin
SARS‐CoV‐2: Severe acute respiratory syndrome coronavirus 2
SGLT2i: Sodium‐glucose cotransporter 2 inhibitors
TF: Serotransferrin
UPP: Urinary peptidomic profiling

## 1 Introduction

As of September 21, 2025, severe acute respiratory syndrome coronavirus 2 (SARS‐CoV‐2) has led to 779 million cumulative COVID‐19 cases and 7.1 million associated deaths, with severe health implications for those who have yet to recover fully [1, 2]. Post‐acute sequelae of SARS‐CoV‐2 infection (PASC) or long COVID is a multisystemic chronic condition, characterized by debilitating symptoms that persist or newly arise in the post‐acute phase of COVID‐19, often presenting with a non‐linear, fluctuating clinical course lasting more than 3 months [3]. Myalgic encephalomyelitis/chronic fatigue syndrome (ME/CFS) can develop after infection with SARS‐CoV‐2, similar to other infections, including those caused by Epstein‐Barr virus (EBV) [4, 5]. The diagnosis of both PASC and ME/CFS currently relies on standardized criteria that are strictly limited to clinical, yet inherently subjective, symptom‐based assessments [6, 7]. On the other hand, measurable biomarkers have the potential to predict disease onset prior to clinical manifestation, reduce diagnostic ambiguity, and enable both early implementation of therapeutic interventions and longitudinal assessment of therapeutic response. Initially based on demographic and clinical parameters, risk assessment for PASC has progressed from demographic and clinical parameters [8] to five self‐reported symptoms in the first week of infection [9] to an interactive recovery prediction tool [10], and more recently, to omics‐based biomarkers requiring further validation [11, 12, 13, 14].

SARS‐CoV‐2 persistence [15], elevated cytokines indicating ongoing inflammation [16], dysregulated complement system [17], and sustained endothelial dysfunction [18, 19, 20] are among the putative pathophysiological mechanisms of PASC, likely engaging in an amplifying feedback loop that drives multi‐organ sequelae [21]. Kidneys are among the organs involved due to their relatively strong expression of the cell surface receptor ACE2 for SARS‐CoV‐2. A decline in the estimated glomerular filtration rate (eGFR) equivalent to a 3.39% reduction from baseline within the first year following infection and a high prevalence of albuminuria among PASC patients have been described [22]. Proximal tubular injury is characterized by low‐molecular‐weight proteinuria with aminoaciduria and has also been documented in hospitalized patients with severe COVID‐19 [23].

Urinary peptidomic profiling (UPP) is known to reveal disease mechanisms of renal and extrarenal origin, as one‐third of urinary proteins are derived from circulating plasma that permeates the glomerular barrier [24]. Capillary electrophoresis‐mass spectrometry (CE‐MS) aided in the discovery of predictive and diagnostic biomarkers through UPP, enabling classifier generation to assess disease mechanisms and progression risk in the subclinical phase of chronic kidney disease [25], diastolic left ventricular dysfunction [26], acute COVID‐19 [27], and more recently, mortality risk in PASC [28].

Given these observations, we hypothesized that the presence of PASC may be inferred from the urinary excretion of smaller proteins and peptides, particularly endogenous peptides, owing to their intricate implication in pathophysiological mechanisms. Regardless of renal origin, detectable alterations in these peptides make urine a valuable medium for detecting and diagnosing multisystemic diseases like PASC. Here, we implemented a 1:1 matched case‐control design, aiming to develop a disease‐specific classifier, titled PASC195, through the analysis of urinary peptidomic data from 50 individuals diagnosed with PASC versus their matched controls.

## 2 Materials and Methods

### 2.1 Study Design and Participants

**FIGURE 1:**
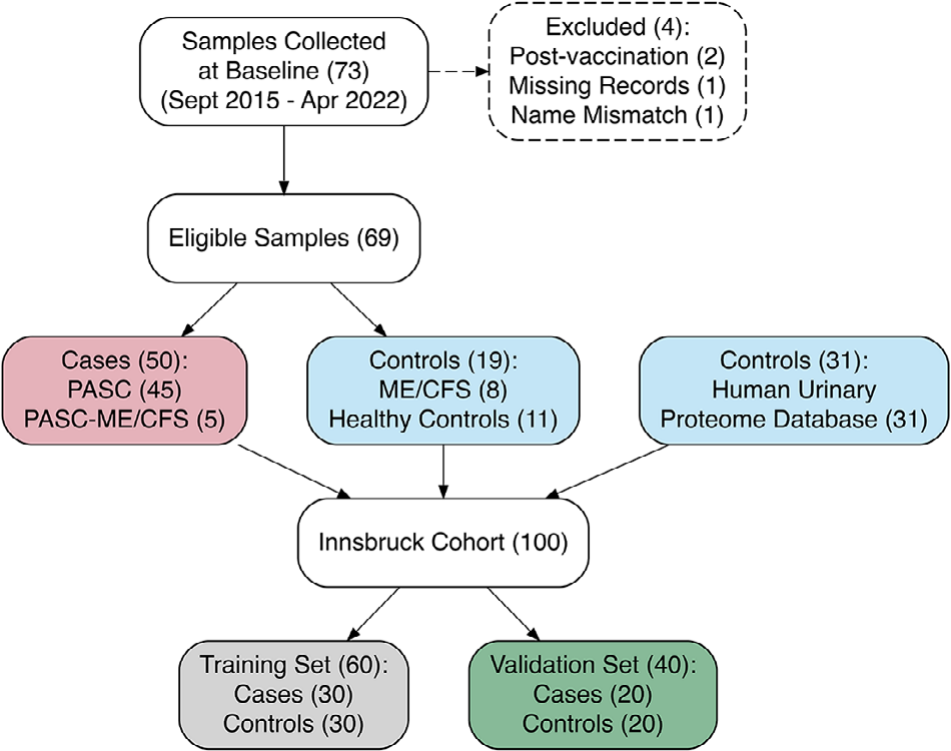
Flowchart for patient recruitment. Flowchart illustrating the selection process for cases and controls in the Innsbruck cohort. Two patients with symptoms arising post‐vaccination without a susceptible or confirmed prior to COVID‐19 infection, one sample missing both electronic medical records and a completed questionnaire, and one case with a name mismatch between the questionnaire and clinical data were excluded from the study. ME/CFS = myalgic encephalomyelitis/chronic fatigue syndrome, PASC = post‐acute sequelae of SARS‐CoV‐2 infection.

The cases and controls in our study population originated from several existing cohorts. Figure 1 presents a flowchart of the cohort used to define the potential biomarker, and for the development and independent validation of the classifier *(Innsbruck cohort)*. The*external prospective cohort of* *previous COVID‐19* *patients* *(UriCoV)* included for external evaluation of the classifier is depicted in Figure SE1.

#### 2.1.1 Innsbruck Cohort

The participants were sourced from the study, Establishment of a Blood, Urine, and Stool Sample Archive for Patients with Fatigue After Infection or Recurrent Infections, conducted at the outpatient clinic for infectious diseases at the Medical University of Innsbruck between September 2015 and April 2022 (EK 1157/2017). The Innsbruck cohort comprised a total of 100 participants. Self‐reported symptoms were quantitatively assessed using the 2003 Canadian Consensus Criteria (CCC) [6] to investigate whether the patients fulfilled the criteria for ME/CFS. The case group consisted of participants diagnosed with PASC, defined as patients with symptoms persisting for 3 months after COVID‐19. Patients with PASC who also met the 2003 CCC [6] for ME/CFS, without a diagnosis of ME/CFS before their SARS‐CoV‐2 infection, were referred to as PASC‐ME/CFS. Case urine samples were collected at a median of 292 days (IQR: 177–412) after COVID‐19 diagnosis. The control group was selected from healthy individuals and those with non‐COVID‐19‐associated ME/CFS, as verified by electronic medical records (EMRs) that either lacked documentation of previous SARS‐CoV‐2 infection or indicated alternative infectious etiologies. Further controls from the Human Urinary Proteome Database [29] were selected as an extension to 1:1 match the number of cases. The final set of 50 case‐control pairs was randomly assigned to a training set of pairs (*n* = 30) or a validation set (*n* = 20). Demographic and clinical data obtained from the EMRs assisted in matching cases and controls. Age was matched on the calendar year of birth. All participants provided written informed consent and were non‐anuric adults (aged ≥18 years).

Detailed protocols for sample preparation for CE–MS analysis, urinary peptidome analysis, peptide sequencing, and classifier generation are described in the Supporting Methods.

#### 2.1.2 UriCoV Cohort

The design, recruitment, and classification of the *external prospective cohort of previous COVID‐19 patients (UriCoV)* are provided in the Supporting Methods.

### 2.2 Statistical Analysis

A preliminary sample size calculation indicated that 26 case‐control pairs were required to detect a 40% change in the abundance of a single biomarker, with a type I error of 0.05 and 80% power. Chi‐squared and Fisher's exact tests were used to compare categorical variables. Group differences were assessed using the paired *t*‐test and Wilcoxon rank sum test, respectively, using MedCalc software (version 12.1.0.0; MedCalc Software, Mariakerke, Belgium) and R version 4.4.1 (2024‐06‐14, ucrt). The description of the statistical analysis performed to define biomarkers is provided in the Supporting Methods.

ROC curve analysis assessed the discriminative performance of the classifier within each cohort. The true positive fraction (sensitivity) and false positive fraction (1‐specificity) were plotted. The AUC was calculated, and an optimal threshold was established using the Youden index. Exact binomial calculations were used to determine 95% confidence intervals (CI). Symptom duration was measured as the interval between the reported onset of the initial acute COVID‐19 infection and the completion of the questionnaire, at which point urine samples were collected or within a short period thereafter.

### 2.3 In Silico Intervention

A recently described pipeline for an in silico intervention was applied to the defined PASC peptides [30, 31]. The previously described interventions (mineralocorticoid receptor antagonists [MRAs], sodium‐glucose cotransporter 2 inhibitors [SGLT2i], glucagon‐like peptide‐1 receptor agonists [GLP‐1RAs], angiotensin II receptor blockers [ARBs], olive oil, and physical activity) were used both individually and in combination to test which intervention could potentially benefit patients.

### 2.4 Use of Artificial Intelligence

Artificial intelligence‐powered tools leveraging large language models (Research Rabbit, Consensus, and ChatGPT OpenAI GPT‐4) aided in manuscript preparation for literature research, grammar enhancement, and proofreading. All outputs were reviewed and finalized by the authors.

## 3 Results

### 3.1 Study Population

#### 3.1.1 Innsbruck Cohort

In Innsbruck, 73 urine samples were collected from participants between September 2015 and April 2022 (at the time of diagnosis for ME/CFS or PASC or upon admission for healthy controls), of which 69 were used for UPP (50 cases and 19 controls) (Figure 1). Questionnaire data were available for only 58% of the Innsbruck cohort (94% of cases and 42.1% of controls), likely because of secondary extension of the control group and non‐participation among asymptomatic controls. The potential nonresponse bias was mitigated by incorporating detailed clinical diagnostic information obtained from EMRs. This ensured that group allocation was based not only on self‐reported symptoms but also on clinician‐confirmed diagnoses, thereby supporting the robustness of case‐control classification. Further datasets of sex‐ and age‐matched individuals were extracted from the Human Urinary Proteome Database. The sample characteristics of the Innsbruck cohort are presented in Table 1.

**TABLE 1:** Characteristics—Innsbruck cohort.

	Overall	Training set			Validation set		
Variable	*N* ^a^ = 100	Case *N* ^a^ = 30	Control *N* ^a^ = 30	*P* value	Case *N* ^a^ = 20	Control *N* ^a^ = 20	*p* value
**Age, yr**	45 ± 12	47 ± 11	45 ± 12	0.5^b^	41 ± 12	48 ± 14	0.067^b^
**Sex**				0.8^c^			0.5^c^
Female	71 (71%)	23 (77%)	21 (70%)		15 (75%)	12 (60%)	
**PASC195 score**	0.18 (−0.86 to 0.74)	0.87 (0.43–0.98)	−0.79 (−0.95 to −0.34)	<0.001^d^	0.67 (0.41–0.84)	−0.91 (−0.96 to −0.84)	<0.001^d^
**ME/CFS**	13 (13%)	3 (10%)	4 (13%)	>0.9^c^	2 (10%)	4 (20%)	0.7^c^
**Hypertension**	16 (16%)	3 (10%)	6 (20%)	0.5^c^	2 (10%)	5 (25%)	0.4^c^
**Use of RAS blockers**	4 (4.0%)	3 (10%)	0 (0%)	0.2^c^	0 (0%)	1 (5.0%)	>0.9^c^
**Cancer**	1 (1.0%)	1 (3.3%)	0 (0%)	>0.9^c^	0 (0%)	0 (0%)	>0.9^c^
**Type 2 diabetes mellitus**	6 (6.0%)	3 (10%)	0 (0%)	0.2^c^	2 (10%)	1 (5.0%)	>0.9^c^
**BMI, kg/m^2^**	23.1 (20.8–27.4)	21.0 (18.9–26.8)	23.1 (21.6–25.6)	0.2^d^	25.9 (21.8–28.2)	23.7 (21.3–27.9)	0.5^d^
**Body mass index ≥30 kg/m^2^**	10 (15%)	4 (24%)	2 (11%)	0.4^c^	3 (23%)	1 (5.9%)	0.3^c^
**SBP, mmHg**	126 ± 22	122 ± 21	129 ± 26	0.4^b^	128 ± 20	125 ± 19	0.7^b^
**DBP, mmHg**	79 ± 13	81 ± 15	78 ± 13	0.5^b^	77 ± 14	79 ± 8	0.6^b^
**eGFR, mL/min/1.72 m^2^**	92 ± 16	91 ± 17	94 ± 13	0.5^b^	94 ± 18	87 ± 18	0.2^b^

Abbreviations: BMI = body mass index, DBP = diastolic blood pressure, eGFR = estimated glomerular filtration rate (2021 CKD‐EPI), IQR = interquartile range, ME/CFS = myalgic encephalomyelitis/chronic fatigue syndrome, RAS = renin‐angiotensin system, SBP = systolic blood pressure, SD = standard deviation.

^a^ Mean ± SD; *n* (%); Median (IQR25–IQR75).

^b^ *t* test.

^c^ Fisher's exact test.

^d^ Wilcoxon rank sum test.

The mean age of the participants was 45.3 years, and 71% were female. Comorbidities included type 2 diabetes mellitus (*n* = 6), overweight (*n* = 10), cancer (*n* = 1), and hypertension (*n* = 16), with four patients being on renin‐angiotensin system (RAS) blockade therapy. One patient had both diabetes and hypertension. Of the eight non‐COVID‐19 ME/CFS patients, two had documentation of chronic or previous EBV infection, one patient had both EBV and Toxocara infection, one had documented Ehrlichiosis, and four were classified as having an unclear etiology. At baseline, 13% of the Innsbruck cohort fulfilled the CCC for ME/CFS [6], compared to 10% of those with PASC.

### 3.2 Classifier Generation and Discriminatory Power of PASC195

#### 3.2.1 Innsbruck Cohort

**FIGURE 2:**
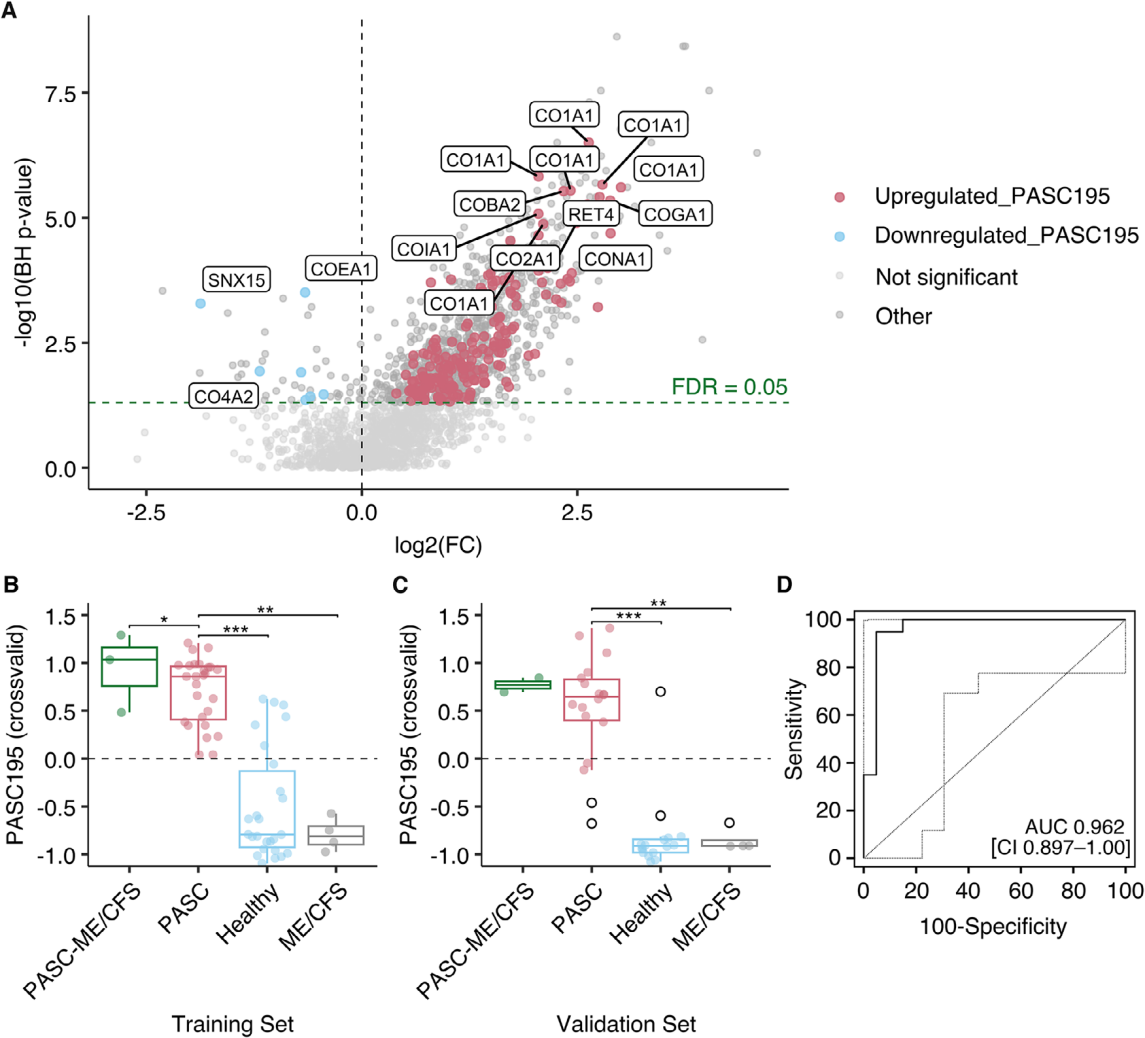
Classifier generation and performance of PASC195. Panel A: Volcano plot displaying log2 fold change (FC) in peptide abundance. FC was calculated as the mean peptide abundance within cases divided by the mean in the control group. The horizontal line indicates the significance threshold (FDR = 0.05). Red dots represent significantly upregulated peptides in PASC195 and blue dots represent significantly downregulated peptides. Light gray indicates non‐significant peptides and gray other peptides. Panel B: PASC195 classification scores of the training set are shown as box‐whisker plots for PASC‐ME/CFS (*n* = 3), PASC (*n* = 27), healthy controls (*n* = 26), and non‐COVID‐19 ME/CFS (*n* = 4). Pairwise comparisons between groups were conducted using the Wilcoxon rank sum test. *p < 0.05, **p < 0.01, and ***p < 0.001 (BH‐corrected). Panel C: PASC195 classification scores of the validation set are shown as box‐whisker plots for PASC‐ME/CFS (*n* = 2), PASC (*n* = 18), healthy controls (*n* = 16), and ME/CFS within the control group (*n* = 4). Black circles indicate outliers. Panel D: Receiver Operating Characteristic (ROC) curve visualizing the performance of PASC195 in distinguishing between cases (*n* = 20) and controls (*n* = 20) within the validation set. The curve plots the sensitivity against 100 ‐ specificity, achieving an AUC of 0.962 (95% CI 0.897–1.00; *p* < 0.0001). The bold black line represents the ROC curve. The gray dotted lines represent 95% confidence intervals. ME/CFS = myalgic encephalomyelitis/chronic fatigue syndrome, PASC = post‐acute sequelae of SARS‐CoV‐2 infection.

Training data from the Innsbruck cohort, including 30 cases (27 PASC, 3 PASC‐ME/CFS) and 30 controls (26 healthy controls, 4 non‐COVID‐19 ME/CFS), were used to define the PASC‐associated urinary peptides. Of the 2034 peptides detected in the urine peptidome analysis, 375 differed significantly in abundance (Figure 2, Panel A). Amino acid sequences could be obtained for 243 peptides, which were further subjected to a take‐one‐out process. This refinement resulted in a final pool of 195 peptides used to generate a classifier, PASC195, consisting of 187 upregulated and eight downregulated peptides. Of these, 172 (88.2%) were aligned to collagen alpha, with 132 (76.7%) linked to collagen alpha‐1 and 29 (16.9%) to collagen alpha‐2 chains. Other peptides included fragments of alpha‐1‐antitrypsin (A1AT), antithrombin‐III (AT III), apolipoprotein A‐II (APOA2), apolipoprotein A‐IV (APOA4), basement membrane‐specific heparan sulfate proteoglycan core protein (PGBM), beta‐2‐microglobulin (B2M), calreticulin (CALR), CD99 antigen, fibrinogen alpha (FIBA) and beta (FIBB), serotransferrin (TF), protein S100‐A9, and mannan‐binding lectin serine protease 2 (MASP2), among others. Among the upregulated peptide sequences, the majority were associated with collagen alpha‐1(I) (CO1A1) (*n* = 50), collagen alpha‐1(III) (CO3A1) (*n* = 29), and collagen alpha‐2(I) (CO1A2) (*n* = 13). Downregulated peptides were fragments of collagen alpha‐2(IV) (CO4A2) (*n* = 2), collagen alpha‐1(XIV) (COEA1) (*n* = 1), clathrin (CLCB) (*n* = 1), glycogen synthase kinase‐3 alpha (GSK3A) (*n* = 1), and Golgin subfamily A member 6‐like protein 2 (GOLGA6L2) (*n* = 1), among others.

The median cross‐validated score in the case group was 0.74 (IQR: 0.44–0.96) and in the controls, −0.86 (IQR: −0.94 to −0.61), respectively. PASC195 achieved an AUC of 0.949 (95% CI 0.900–0.998; *p* < 0.0001) when estimated using complete leave‐one‐out cross‐validation on the training set (Figure 2, Panel B). In the independent validation set of 40 individuals (20 cases and 20 controls), the classifier effectively distinguished patients with PASC from controls, yielding an AUC of 0.962 (95% CI 0.897–1.00; *p* < 0.0001) (Figure 2, Panels C and D). The sensitivity and specificity of 95% were at a cut‐off of −0.596 (positive and negative predictive value of 95%), supporting the model's robustness and predictive accuracy in an independent dataset. The peptidome profile of PASC195 is shown in Figure 3 and the Supporting Results (SE3).

FIGURE 3Urinary peptidomics profiling (UPP) of the training set by subgroup (PASC195). UniProt symbols corresponding to each peptide are displayed along the y‐axis. Log2‐transformed peptide signal intensities were Z‐normalized and hierarchically clustered within four subgroups: PASC‐ME/CFS, PASC, healthy controls, and non‐COVID‐19 ME/CFS. The start and end positions of the amino acids are indicated in parentheses. Panel A displays the top 50 significant collagenous peptides, and panel B illustrates all the non‐collagenous peptides of the PASC195 classifier. See Supporting Material for the complete peptidome profile. Green, pink, and gray indicate higher signal intensities than mean peptide abundance, lower signal intensities, and missing values, respectively. ME/CFS = myalgic encephalomyelitis/chronic fatigue syndrome, PASC = post‐acute sequelae of SARS‐CoV‐2 infection.

**Figure:**
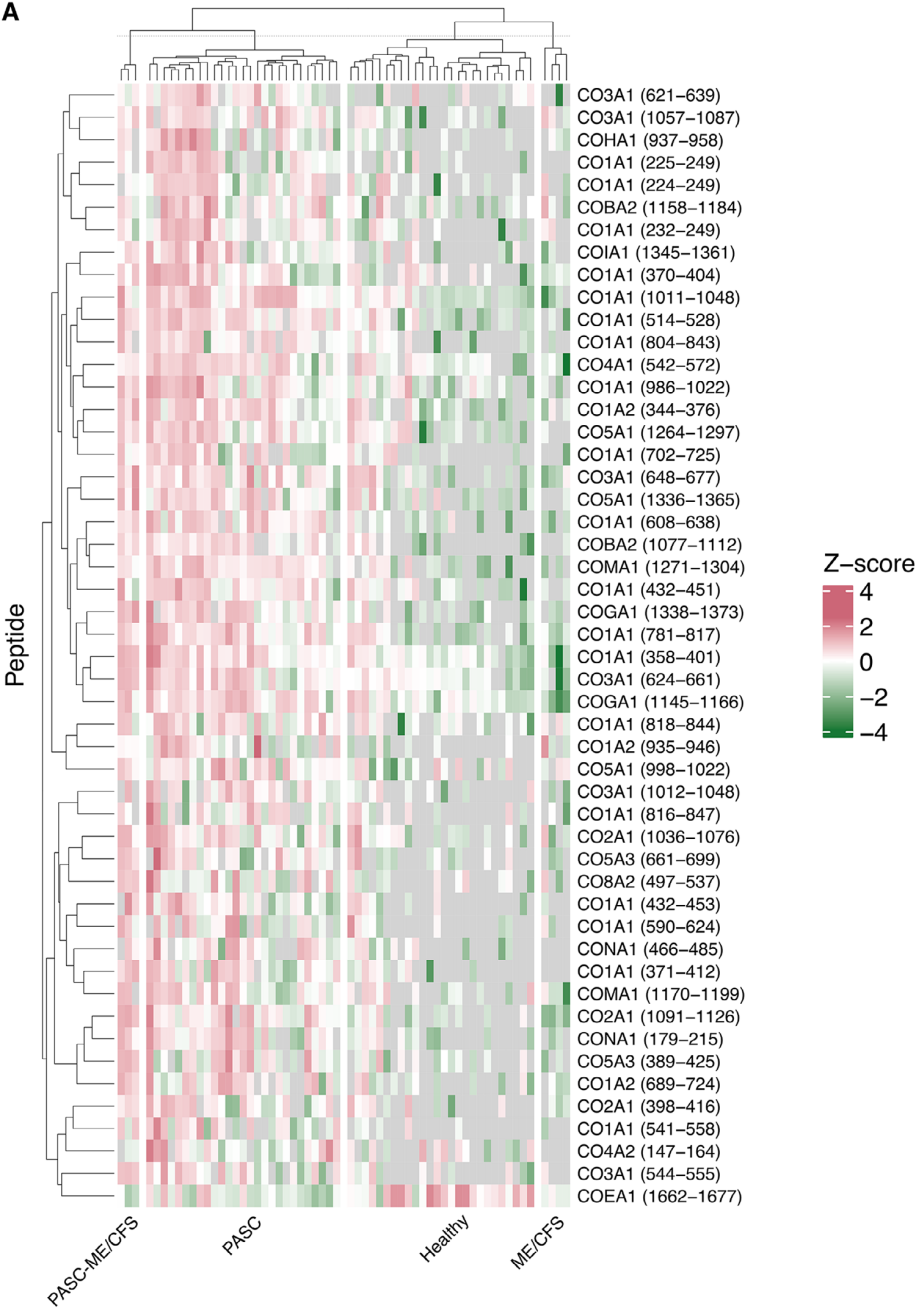

**Figure:**
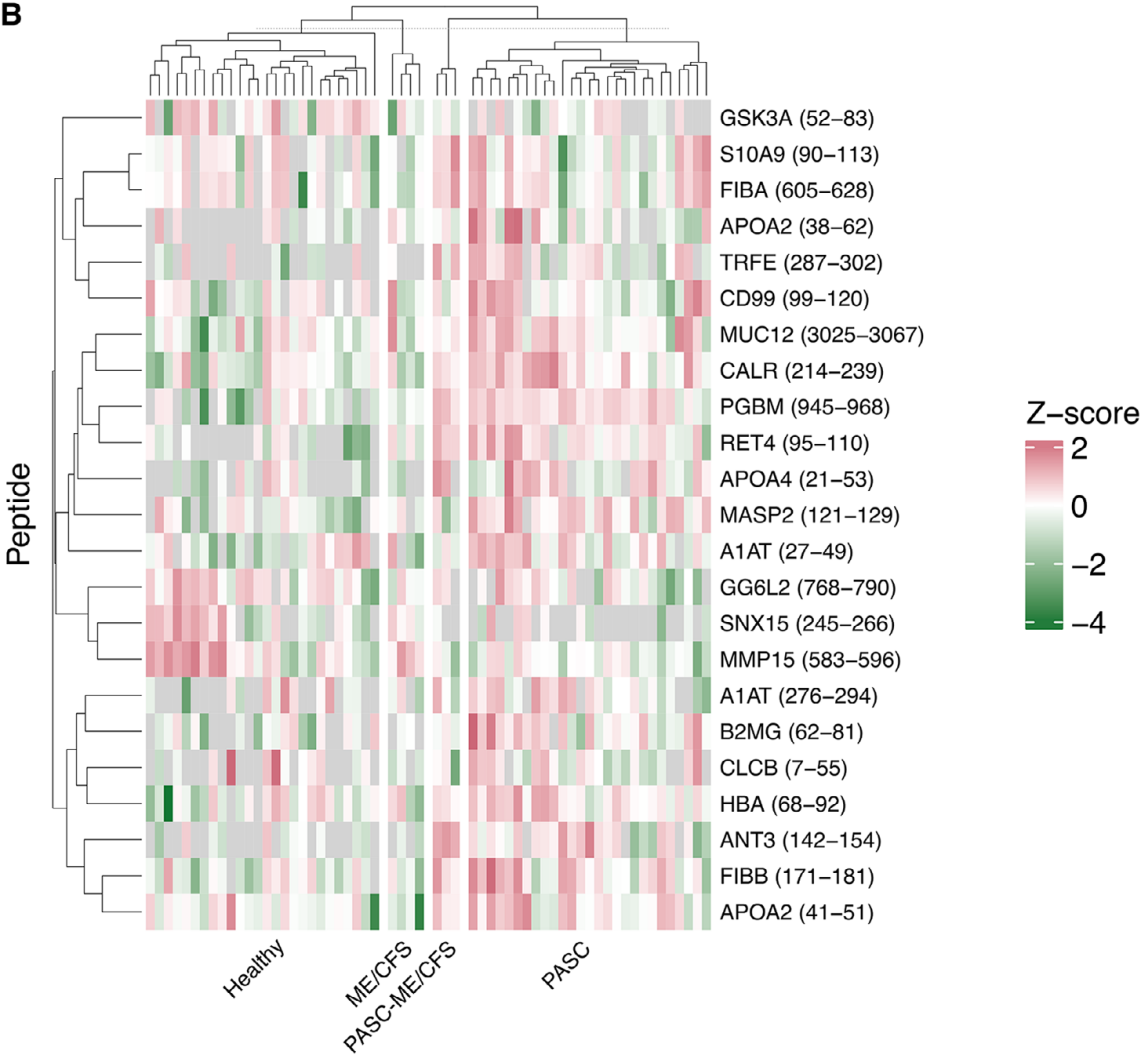

#### 3.2.2 UriCoV Cohort

The classifier's performance was evaluated in the multicenter prospective UriCoV cohort. Sensitivity analysis yielded no statistically significant difference (*p* = 0.1492) between cases (*n* = 34) and controls (*n* = 40). Detailed findings are included in the Supporting Results.

### 3.3 In Silico Intervention

As no specific therapeutic approach has yet demonstrated consistent efficacy in PASC, we employed our recently described algorithm to simulate in silico responses to six interventions (MRAs, SGLT2i, GLP‐1RAs, ARB, olive oil, and physical activity) [30, 31]. Applied to PASC patient data from the Innsbruck cohort, the algorithm estimated heterogeneous individual responses (Supporting Table S2). The average predicted improvement in the PASC195 scores was 0.131 ± 0.044 when the most suitable intervention was assigned to each patient. Physical activity, MRAs, and GLP‐1RAs, in particular, were predicted to be beneficial in most cases.

### 3.4 Clinical Symptoms in Case and Control Subgroups and Their Association With PASC195 Score

**FIGURE 4:**
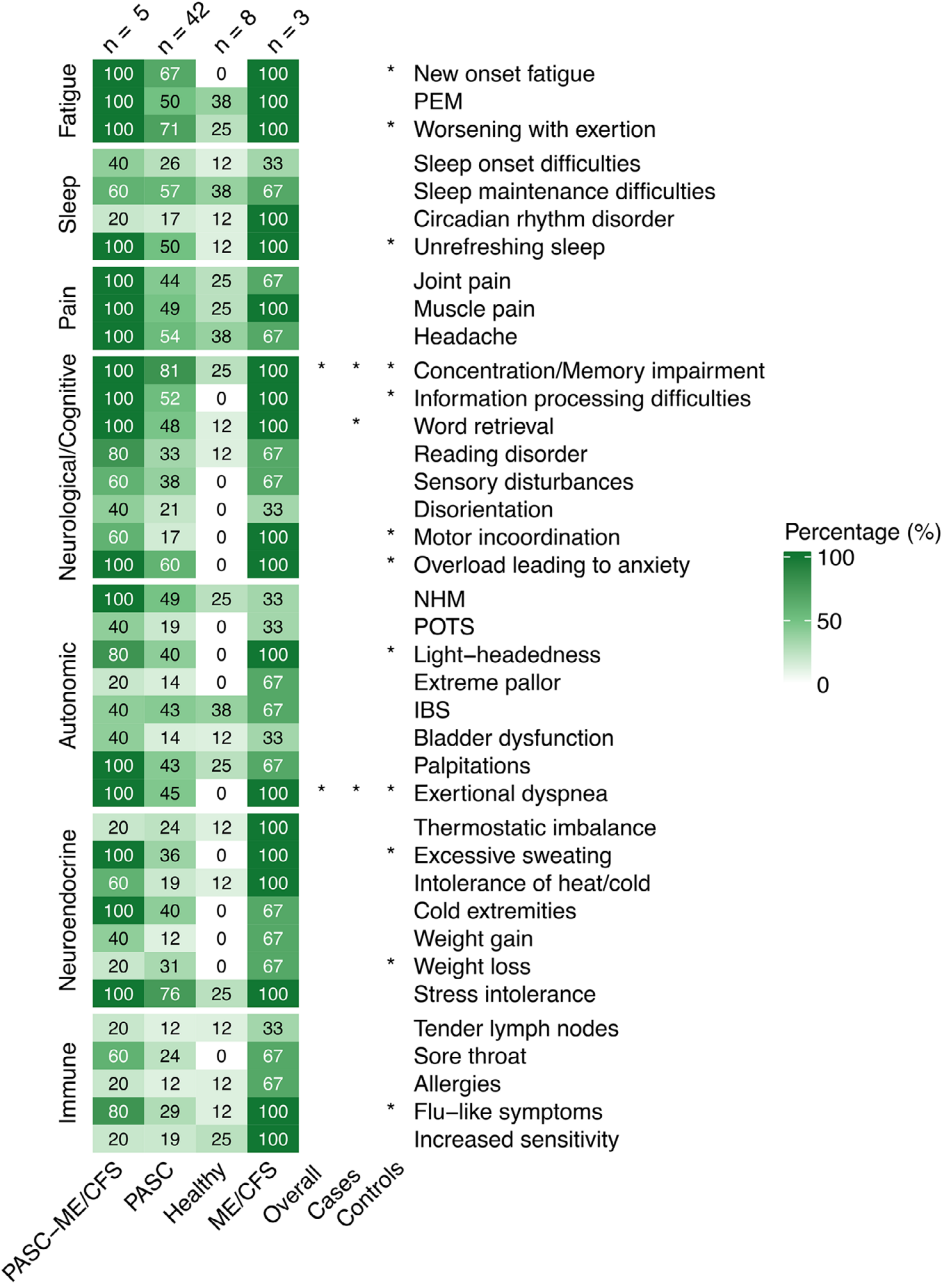
Symptom profile by diagnosis and associations with PASC195 scores. Heatmap illustrating response distributions in percentages (%) for four subgroups (PASC‐ME/CFS, PASC, healthy controls, and non‐COVID‐19 ME/CFS) by manifestation. The color intensity increases by percentages. The sample sizes of questionnaire respondents are shown in parentheses. Significant differences in the classification scores between symptomatic and asymptomatic patients are marked with asterisks (*) in the overall cohort, cases, and controls (Wilcoxon rank sum test). *p* values were not corrected for multiple testing. *p < 0.05, **p < 0.01, and ***p < 0.001. IBS = irritable bowel syndrome, ME/CFS = myalgic encephalomyelitis/chronic fatigue syndrome, NHM = delayed postural hypertension or neurally mediated hypotension, PASC = post‐acute sequelae of SARS‐CoV‐2 infection, PEM = post‐exertional malaise, POTS = postural orthostatic tachycardia syndrome.

Self‐reported symptoms at baseline [median duration: 300 days (IQR: 169–384)] were assessed using the CCC [6], which categorizes the diagnostic criteria for ME/CFS into seven domains (Fatigue, Sleep dysfunction, Pain, Neurological/Cognitive manifestations, Autonomic manifestations, Neuroendocrine manifestations, Immune manifestations) and 38 symptoms. Concentration impairment and short‐term memory consolidation were the most frequently reported symptoms overall (74.1%), and among cases (80.9%, a median duration of 303 days [IQR: 186–426]). Tender lymph nodes were the least frequently reported symptom (13.8%), with a median duration of 325 days (IQR: 220–334). Detailed response distributions for each symptom are presented in Figure 4 and Table S3.

Significantly higher PASC195 scores were found in the presence of concentration impairment and short‐term memory consolidation (*r* = 0.27, *p* = 0.044), and exertional dyspnea (*r* = 0.26, *p* = 0.048) among all questionnaire respondents, as shown in Figure 4. In a subgroup analysis of only cases, significant differences were found in word retrieval (*r* = 0.31, *p* = 0.036), concentration impairment and short‐term memory consolidation (*r* = 0.32, *p* = 0.031), and exertional dyspnea (*r* = 0.31, *p* = 0.032), with each symptom associated with higher scores compared to cases without the respective symptom. However, due to the exploratory nature of this analysis, these calculations were not corrected for multiple testing.

## 4 Discussion

In the absence of consensus‐based diagnostic models derived from an understanding of disease mechanisms, we aimed to identify urinary peptides hinting at PASC pathophysiology using a data‐driven approach. Although direct comparisons between our urinary peptidome data and previously reported plasma or serum datasets are not feasible due to differences in sample types, methodologies, and data processing, we observed analogous peptide patterns. We found molecular signatures suggestive of collagen dysregulation, prothrombotic events, and potential signs of endothelial dysfunction reflected in the urine peptides, consistent with similar findings reported in previous omics‐based PASC studies using serum or plasma as derivatives [12, 17, 32, 33, 34, 35]. Second, we developed a novel classifier, PASC195, comprising 195 modulated peptides that significantly differed from the UPP of individuals without PASC, including those with other post‐acute infectious syndromes progressing to ME/CFS. Third, an exploratory analysis of classification scores and 38 symptoms linked PASC195 to neurocognitive (word retrieval deficits, concentration impairment, and short‐term memory consolidation), as well as autonomic manifestations (exertional dyspnea).

UPP likely captures peptides originating from both plasma and the renal or urinary tract; however, assigning a specific origin to individual peptides remains technically complex. The dysregulated peptides observed in PASC may arise from a combination of systemic and renal processes, including inflammation, endothelial dysfunction, and altered proteolysis. Although direct quantification of proteolytic activity was not feasible, the observed associations of PASC195 scores with neurocognitive and autonomic symptom domains indicate that the urinary peptidome may mirror multisystem pathophysiological processes characteristic of PASC.

A shift in collagen homeostasis is a representative feature of PASC195, as the majority of dysregulated peptide sequences stem from CO1A1, like the COV50 classifier, a urinary proteomic biomarker comprising 50 peptides. COV50 has been associated with predicting adverse outcomes during the acute phase and a predisposition to death even in the absence of COVID‐19 infection [36]. The CO1A1 downregulation in COV50 has been attributed to attenuated collagen degradation within the extracellular matrix, likely leading to increased fibrosis [36]. In contrast to COV50, CO1A1 fragments were upregulated in PASC195, with no overlap in peptide sequences between the two classifiers. Another shared component of COV50 and PASC195 is the presence of the degradation products of A1AT, which were reported to be upregulated in COVID‐19 [27], and here in PASC, respectively. A1AT is an inhibitor of serine proteases such as TMPRSS‐2, which facilitates SARS‐CoV‐2 binding to the ACE2 receptor via spike protein activation, and also acts as an acute‐phase protein with anti‐inflammatory and vascular protective effects [37]. Its higher abundance could imply an amplification loop of ongoing inflammation or endothelial injury in PASC.

Confirming earlier findings from a study on the plasma proteome in patients with PASC [12, 34, 35], we detected upregulation of fibrinogen subunits FIBA and FIBB in the urine peptidome. Notably, the same study demonstrated decreased FIBA and FIBB levels in pellet fractions. One possibility is that this reduction results from abnormalities in proteolytic activity or accelerated degradation, which may manifest as a consequent enrichment of fibrinogen subunits in urine. A recent serum proteome study identified elevated levels of FIBB alongside other coagulation cascade components (factor XI, protein C, and heparin cofactor II), as well as decreased AT III (linked by the authors to increased cleavage activity, a potential explanation for our observations regarding its abundance) 6 months post‐infection [17]. Because fibrin is involved not only in platelet aggregation but also in preventing IFN‐γ‐induced hemorrhage and triggering immune responses [38], these findings should not be confined to a hypercoagulable state alone. Disruptions in iron homeostasis and inflammatory anemia were described as early indicators of PASC in a longitudinal cohort study [39]. Serum iron and transferrin saturation persisted at significantly lower levels for up to 9 months after severe/critical COVID‐19, consistent with our observation of high prevalence of impaired cognitive function, fatigue, and upregulation of serum transferrin.

Siwy et al. reported CD99 antigen depletion specific to severe acute COVID‐19 in a urinary peptidome study [40]. Given its role in leukocyte extravasation and T‐cell integration [38], a compensatory immune response may account for its upregulation in PASC. Calprotectin (S100A8/A9) has been shown to correlate with disease severity and increased mortality risk in acute COVID‐19 [12, 32, 41]. In line with immune dysregulation, we observed a higher abundance of peptides derived from calprotectin subunits (S100A9). Another remarkable finding was the overexpression of PGBM, a component of basal membranes, including the glomerular basement membrane, possibly linked to increased peptide leakage or systemic alterations in endothelial barrier function.

Following our previously published approach for in silico intervention in CKD [31], we applied the algorithm to the PASC patient data described here. This exploratory analysis, motivated in part by the lack of established therapies for PASC, identified exercise, MRAs, and GLP‐1RAs as potentially beneficial for the majority of patients. Although this approach does not constitute evidence of efficacy and certain interventions (e.g., exercise) may not be appropriate for all individuals, it outlines a potential path forward for targeted interventions.

Despite the fluctuating course of PASC, symptoms have often been observed to resolve or improve over the subsequent years [42]. Urine sampling in the UriCoV cohort occurred approximately 3 years after the initial SARS‐CoV‐2 infection, compared to 10 months post‐infection in the Innsbruck cohort. In UriCoV, the presence of ongoing or prior symptoms at the time of sampling was assessed through a structured self‐report questionnaire, primarily reflecting perceived symptom burden, as clinical evaluation was not part of the study protocol, whereas in Innsbruck, case status was established through clinician‐confirmed diagnosis. The high proportion of reported cases in UriCoV suggests that differences in questionnaire design and reliance on self‐reported data may have contributed to diagnostic variability, potentially affecting case classification. Unlike Innsbruck participants, UriCoV subjects were not routinely recruited from an infectious disease outpatient clinic, which may have introduced selection bias related to symptom perception and reporting. Collectively, these factors, recognized only during the study, suggest that at the time of sampling, the clinical presentation of participants classified as PASC in UriCoV likely differed in severity or disease stage from those examined 10 months post‐infection in Innsbruck. Cohort‐specific differences in case and control definitions, symptom burden, and participant selection likely contributed to variability in urinary peptidomic profiles and precluded validation of the PASC195 classifier in this independent cohort, thereby limiting its generalizability.

Moreover, including individuals with other post‐acute infectious syndromes as a separate comparator group, as well as conducting sub‐cohort analyses by sex, age, or other clinical parameters, would aid in identifying potential subgroup‐specific associations and in delineating PASC‐specific molecular signatures. However, the limited sample size did not permit such analyses, as the small number of participants in each subgroup would have substantially reduced statistical power and reliability. Future investigations with larger and more heterogeneous populations should address potential sex‐ or age‐specific differences in urinary peptidomic alterations associated with PASC. Furthermore, integrating urine, plasma, and tissue‐level proteomics across diverse post‐infectious cohorts, under standardized diagnostic protocols, will be essential to validate the PASC195 classifier and elucidate underlying mechanisms. Taken together, these inconsistencies underscore the urgent need for harmonized recruitment and phenotyping procedures to enable cross‐cohort comparability and support validation of urinary biomarkers in broader PASC populations.

In summary, we present a PASC‐associated urinary peptidome profile that reveals cross‐sectional variations in collagen homeostasis, inflammatory responses, and hemostatic processes distinct from non‐COVID‐19 ME/CFS and healthy controls, suggesting differences in disease pathogenesis. Our findings may not only hold clinical and predictive relevance by supporting molecular differentiation of PASC but also provide an objective biological correlate that complements patient‐reported outcomes. These findings lay the groundwork for precise detection and, through in silico intervention, a potential pathway toward the treatment of PASC.

## Author Contributions

**Dilara Gülmez**: formal analysis, investigation, data collection, writing – original draft, and visualization. **Justyna Siwy**: conceptualization, methodology, formal analysis, investigation, resources, data collection, writing – review and editing, visualization, supervision, and project administration. **Katharina Kurz**: conceptualization, investigation, resources, data collection, writing – review and editing, and supervision. **Ralph Wendt**: conceptualization, investigation, data collection and resources. **Miroslaw Banasik**: conceptualization, investigation, data collection, resources and writing – review and editing. **Björn Peters**: conceptualization, investigation, data collection, resources, and writing – review and editing. **Emmanuel Dudoignon**: investigation, resources, data collection, writing – review and editing. **Elena Nowacki**: investigation, data collection. **Amelie Kurnikowski**: writing – review and editing. **Sebastian Mussnig**: writing – review and editing. **Simon Krenn**: writing – review and editing. **Samuel Gonos**: data collection. **Judith Löffler‐Ragg**: writing – review and editing. **Günter Weiss**: writing – review and editing. **Harald Mischak**: conceptualization, methodology, formal analysis, investigation, resources, writing – review and editing, supervision, and funding acquisition. **Manfred Hecking**: conceptualization, investigation, resources, writing – review and editing, supervision, and funding acquisition. **Eva Schernhammer**: writing – review and editing, supervision. **Joachim Beige**: conceptualization, investigation, resources, writing – review and editing, and supervision.

## Funding

This project was supported by the Federal Ministry of Health (BMG) via grant number 2523FSB114; by the German Ministry for Education and Science (BMBF) via grant 01KU2309; by the State of Tyrol via grant number GZ 75759; by Fisser Bergbahnen through a benefit gala donation (2022) under the project name “*Projekt ME/CFS‐Forschung*”; by the State of Tyrol and the WE&ME Foundation via grant number GZ 86686; by the Sweden's innovation agency (VINNOVA) via grant 2022‐00542; by the National Centre for Research and Development (NCBR) via grant number: PerMed/V/80/UriCov/2023; by the Austrian Science Fund (FWF) via Project number I 6464 and by the French National Research Agency (ANR)—under the grant ANR‐22‐PERM‐0014. The funders were not involved in the study design, data collection, data analysis, interpretation of results, or manuscript preparation.

## Ethics Statement

The study was approved by the Ethics Committee of the Medical University of Innsbruck (Innsbruck, Austria; EK 1157/2017) and the German‐Saxonian Board of Physicians (Dresden, Germany; EK‐BR‐88/20.1).

## Consent

Informed consent was obtained from all subjects involved in this study.

## Conflicts of Interest

H.M. is the co‐founder and co‐owner of Mosaiques Diagnostics. J.S. is employed by Mosaiques Diagnostics GmbH. The other authors declare no conflicts of interest.

## Supporting information




**Supporting Information File 1**: pmic70074‐sup‐0001‐SuppMat.docx

## Data Availability

Patient data cannot be made publicly available due to GDPR restrictions. The datasets used and/or analyzed during the current study are available from the corresponding author on reasonable request. Proposals will be reviewed and approved by the authors with scientific merit and feasibility as the criteria. After approval of a proposal, data can be shared via a secure online platform after signing a data access and confidentiality agreement. Data will be made available for a maximum of 5 years after a data sharing agreement has been signed.
